# CD1: From Molecules to Diseases

**DOI:** 10.12688/f1000research.12178.1

**Published:** 2017-10-30

**Authors:** D. Branch Moody, Sara Suliman

**Affiliations:** 1 Division of Rheumatology, Immunology Allergy, Department of Medicine, Brigham and Women’s Hospital, Harvard Medical School, Boston, Massachusetts, USA

**Keywords:** CD1, antigen display, TCR, T cell receptor

## Abstract

The human cluster of differentiation (CD)1 system for antigen display is comprised of four types of antigen-presenting molecules, each with a distinct functional niche: CD1a, CD1b, CD1c, and CD1d. Whereas CD1 proteins were thought solely to influence T-cell responses through display of amphipathic lipids, recent studies emphasize the role of direct contacts between the T-cell receptor and CD1 itself. Moving from molecules to diseases, new research approaches emphasize human CD1-transgenic mouse models and the study of human polyclonal T cells *in vivo* or *ex vivo* in disease states. Whereas the high genetic diversity of major histocompatibility complex (MHC)-encoded antigen-presenting molecules provides a major hurdle for designing antigens that activate T cells in all humans, the simple population genetics of the CD1 system offers the prospect of discovering or designing broadly acting immunomodulatory agents.

## Molecules to disease

Scientific inquiry into major histocompatibility complex (MHC) and cluster of differentiation (CD)1 antigen presentation to T cells developed in separate, nearly anti-parallel tracks. Key biological roles of thymic “T cells” were known fifty years ago ^1^. The molecular identity of antigens and an understanding of how they are displayed by MHC proteins unfolded over the following three decades. Starting with clear evidence for a role of T cells in tissue transplantation and donor-restricted recognition of intracellular viruses ^2, 3^, subsequent experiments mapped MHC genes, detected MHC protein heterodimers, and solved the tricky business of proving that antigens bind within (rather than beside) MHC proteins ^4^.

Whereas MHC I and II research followed a disease-to-molecules trajectory, CD1 research is unfolding as a molecules-to-disease story. At the outset, CD1 proteins were inferred to be antigen-presenting molecules on the basis of their homology to MHC I genes ^5^, association with beta-2-microglobulin ^6^, and restricted expression on professional antigen-presenting cells. Experimental biologists proved a role for CD1 in antigen presentation through landmark discoveries relating to identification of lipid antigens for T cells ^7, 8^, identification of CD1d as the target receptor for natural killer T (NKT) cells ^9^, and generating crystallographic evidence that T-cell receptors (TCRs) directly contact and discriminate the structures of CD1-lipid complexes ^10^.

This brief history is instructive because it explains why the molecular understanding of lipid antigen recognition greatly exceeds knowledge of the roles of CD1-reactive T cells in disease. Nevertheless, the CD1 system is broadly conserved in mammalian evolution, and many studies now show that CD1-reactive T cells are common in human blood ^11^. Through new animal models and the recent development of human CD1a, CD1b, and CD1c tetramers, insight into the roles of CD1 and lipid antigens in human immunology are now emerging. The roles of CD1d, alpha-galactosyl ceramides and NKT cells *in vivo* have been thoroughly reviewed elsewhere ^12^, so this summary focuses on the relatively overlooked and functionally distinct roles of the CD1a, CD1b, and CD1c antigen display systems. Here, we explain CD1-lipid-TCR interactions, highlight two recent examples of antigen recognition that diverge from accepted models, and summarize recent studies of disease-focused work in humans and human transgenic mice.

## Head group recognition model

**Figure 1.**
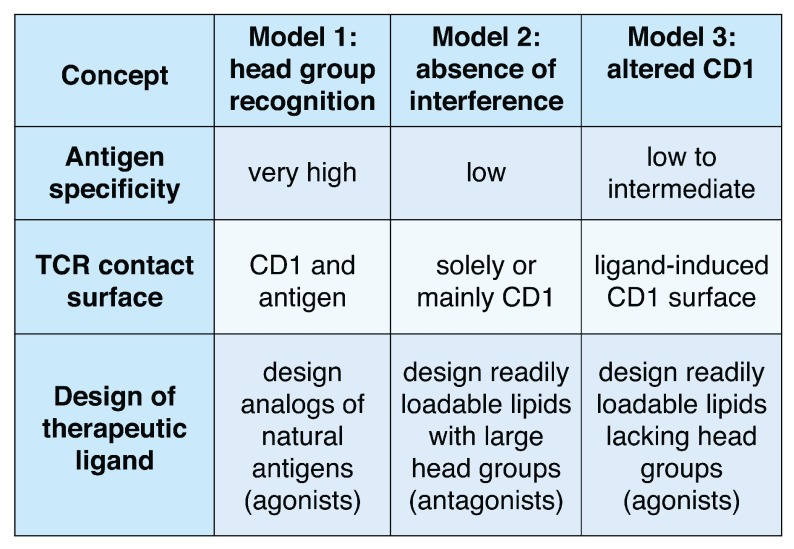
Three general models for CD1–lipid–T-cell receptor ternary interactions that highlight possible approaches to the design of therapeutic ligands. TCR, T-cell receptor.

The earliest analysis of CD1 sequences with Kyte-Doolittle plots predicted that CD1 proteins would fold to form hydrophobic clefts ^13^. Crystal structures of CD1a ^14, 15^, CD1b ^16^, CD1c ^17^, and CD1d ^18, 19^ have subsequently demonstrated that each of these proteins has hydrophobic clefts and pockets of different sizes, which accommodate the aliphatic hydrocarbon chains present in lipid, glycolipid, phospholipid, or lipopeptide antigens. Structure-function studies of these antigens initially pointed to a model in which the T-cell recognition was precisely controlled by the carbohydrate, peptide, or other polar elements present in the head group moiety, but the lipidic moieties could be changed with only a limited effect on T-cell activation ^20– 22^. Confirming these early functional results, later crystallographic studies of CD1d-glycolipid-TCR ^10^ and CD1b-glycolipid-TCR ^23^ revealed that the hydrophilic head groups protrude above the cleft, allowing the TCR to contact the head group moiety directly. In some cases, the TCR contacts the head group as it lies on the outer surface of CD1, and in other cases, the TCR induces substantial conformational change to the head group to press or “bulldoze” it down on CD1 ^24– 26^. Collectively, these studies suggested a “head group recognition” model for CD1b and CD1d that emphasizes direct and extensive contact of the TCR with antigen ( Figure 1). These data prove that the TCR can specifically bind to and discern the structure of carbohydrate and other non-peptidic head groups, expanding prior views that TCRs react solely to peptides.

According to the head group recognition model, TCRs predominantly contact a hybrid epitope formed by the outer surface of CD1 and the exposed polar head groups of carried antigens. Because the lipid tails are largely hidden within CD1, natural or experimental alterations do not necessarily abrogate recognition. Altering the lipid anchor can produce no effect ^22^, relatively small effects based on ease of loading ^27^, or indirect effects based on changing the overall configuration of the CD1-lipid complex. For example, longer lipid anchors or lipid side chains can move the head group of CD1b-presented sulfolipids or cause local changes in the surface of CD1d ^28– 32^.

The head group recognition model is similar to basic MHC-peptide-TCR recognition because TCR contact with the MHC-peptide surface explains high specificity of T cells for both the antigen and antigen-presenting molecule ( Figure 1). Because the recognition of most known lipid antigens is very precisely controlled by the structure of the carbohydrate or peptide moieties present on glycolipids or lipopeptides, head group recognition was thought to be the universal mechanism of antigen recognition in the CD1 system. However, recent studies of CD1a and CD1c antigen complexes provide evidence for alternate models: “absence of interference” and “altered CD1”.

## Absence of interference model

Isolation of autoantigens from skin and other tissues demonstrated that self-lipids like squalene could mediate CD1a autoreactivity ^33^. Squalene is a small alkane lipid, which lacks any hydrophilic head group moieties present in the previously described CD1 antigens. Furthermore, studies of a CD1a and squalene-responsive T-cell clone known as BC2 demonstrated cross-reactivity to other small hydrophobic molecules like wax esters and free fatty acids. Thus, the key premise (hydrophilic moieties in the antigen) and the key prediction (fine specificity for the hydrophilic moieties) of the head group recognition model were not fulfilled for CD1a-presented autoantigens. These functional results from T-cell assays invited consideration of a new structural model of CD1 recognition.

Because certain CD1a autoantigens were small relative to previously described antigens and the known molecular volume of CD1 clefts, an idea emerged that they might nest deeply within CD1a and allow recognition by “absence of interference” with the approaching TCR ^33– 35^. The first solved ternary structure of CD1a-lipid-TCR revealed that the TCR solely contacts epitopes on the CD1a protein and completely misses the carried lipids, lysophosphatidylcholine, and fatty acid. A binary structure of CD1a proteins bound to fatty acids and other molecules showed that the lipid density was seated wholly within CD1a, so fatty acids fulfill the original prediction of the absence of interference model. Lysophosphatidylcholine is not fully nested within CD1a, but instead protrudes from the cleft, but does so at an angle that pushed the head group laterally away from the TCR contact area of CD1a ^36^. These studies identified two mechanisms by which lipids could bind CD1a yet fail to interfere with the TCR-CD1a contact: lateral escape or seating below the plane of TCR contact.

Conversely, sulfatide and sphingomyelin, which have large polar head groups, could block recognition of CD1a by CD1a-autoreactive TCRs. In crystal structures of CD1a-sphingomyelin, the sphingomyelin head group substantially disrupted a triad of residues located with the A′ roof of the CD1a protein, which is located under the TCR footprint ^36^. This mechanism of interference involves alteration of CD1a itself. A somewhat different mechanism of interference likely occurs with other CD1 ligands with large head groups. In CD1d-ganglioside D3 complexes, the large head group presumably wedges between TCR and CD1d, creating stearic interference between CD1d and TCR ^37^. Thus, the large head groups *interfere* with the CD1a-TCR interaction by two distinct but related mechanisms.

These studies rule in the absence of interference model for three TCRs in the CD1a system and make some general predictions about TCR contact with lipid and CD1 that are different from the head group recognition model ( Figure 1). For these TCRs, small hydrophobic ligands could function to augment response, regardless of their fine structure, so long as they can efficiently load into CD1a and fail to interfere with TCR contact with CD1a. In contrast, many phospholipids and sphingolipids, which have large head groups, would be expected to block autoreactive TCRs ^38^. Thus, these structural studies raise the possibility that readily loadable lipids with small head groups could be developed as agonists for CD1 autoreactive T cells. Loadable lipids with large head groups might be antagonists. Currently, the absence of interference model is supported by fewer examples than those underlying head group recognition, but it has potential to explain immune surveillance by the high numbers of CD1a-autoreactive T cells circulating in human blood ^38, 39^.

## Altered CD1 model

The first structure of CD1c was solved in complex with a foreign lipid known as mannosyl phosphomycoketide ^17^. This structure revealed that, rather than having a single point of access to the cleft, CD1c has a series of holes or portals, known as the F′ portal, D′ portal, and E′ portal. A recent article by Mansour and colleagues shows that self-cholesteryl esters trigger a major reorganization of CD1c upon binding. The cholesteryl ester triggers a motion in CD1c that is like a Venus flytrap closing on its prey ^40^. The looser structure of CD1c, dominated by three portals, might create a situation in which the dominant effect of lipid binding is the gross alteration of the three-dimensional surface of CD1c, an effect that is in addition to, or instead of, a lipid head group protruding to the surface ^40^.

This “altered CD1” model and the “absence of interference” model both predict that the TCR contacts solely, or mostly CD1 and not lipid. Both models predict lower TCR fine specificity for the structures of the antigens bound ( Figure 1). The latter emphasizes the role of blockers that prevent TCR contact of CD1a ^36^. The former predicts that the overall shape of CD1c changes depending on the presence or absence of lipids bound. Structurally, evidence for “altered CD1” is supported by binary structures of CD1c-lipid ^17, 40^ and functional studies of antigen recognition ^41, 42^. Formal structural proof of this model awaits the solution of ternary structures of CD1c-lipid-TCR.

## Human CD1 tetramers

Tetramers represent a major technical advance in the study of T cells ^43^. Based on the antigen-specific nature of TCR interactions with tetramers of antigen-presenting molecules, antigen-loaded tetramers allow the direct tracking and sorting of individual antigen-specific T cells within complex mixtures of cells in ways that are otherwise not possible ^44^. Whereas mouse and human CD1d tetramers have been available for more than a decade ^45^, human CD1a, CD1b, and CD1c tetramers have only recently been validated ^40, 42, 46– 49^. A technical challenge has been the difficulty in loading hydrophobic lipids into CD1 proteins in aqueous solution. However, once loading has been accomplished for a particular CD1-lipid pair, the non-polymorphic nature of CD1 proteins creates a situation in which CD1 tetramers can be used on any donor without genetic (MHC locus) matching.

In this young field, most studies have focused on mycobacterial ligands, including dideoxymycobactin ^47^, phosphomycoketides ^42, 48^, mycolic acid ^32^, and glucose monomycolate ^49^, in staining T cells from the blood of patients with active or latent tuberculosis. These studies provide clear evidence for polyclonal lipid-reactive T cells in humans and certain aspects of their phenotypes, such as conserved TCRs, CD4 expression, and prominent secretion of tumor necrosis factor alpha (TNFα) and interferon gamma (IFNγ). A missing piece is the need to track CD1-reactive T cells over time during infection to determine whether such cells play a role in immunological memory. 

## Human CD1-transgenic mice

CD1a, CD1b, and CD1c are not expressed in mice and rats ^50^, so tractable small animal models for study of these three CD1 types *in vivo* were limited. However, guinea pigs express CD1a, CD1b, and CD1c ^51^, allowing analysis of mycobacterial infection and vaccination ^52, 53^. Also, transgenic mice expressing human CD1a, CD1b, and CD1c under control of endogenous human promoters show expression patterns similar to those of humans, allowing analysis of mycobacteria-reactive CD1-restricted T cells *in vivo* ^54^. Combining human transgenic CD1 proteins and the responding TCRs, mice co-expressing a CD1b-autoreactive TCR (HJ1) and CD1b displayed intrathymic-positive selection on CD1b-expressing hematopoietic cells and peripheral activation of HJ1 transgenic T cells in a CD1b-dependent manner ^55^. In response to CD1b expression and interleukin-12 (IL-12) exposure, HJ1 T cells secreted IFNγ and IL-17, contributing to local inflammation and tissue pathology. However, HJ1 T cells also conferred some anti-microbial control against listeria challenge ^55^, suggesting that they can be either pathogenic or protective, depending on the context.

CD1b is an attractive candidate for surveillance of pathogen-derived lipid antigens given its potent upregulation *in vivo* in response to bacteria ^56^, IL-1 ^57^, or granulocyte-macrophage colony-stimulating factor (GM-CSF). Moreover, CD1b shows particularly efficient trafficking through acidic endosomal and lysosomal compartments, which are the usual sites for accumulation of exogenous bacteria or foreign lipid antigens ^58, 59^. Expression of a CD1b-restricted, mycolic acid–specific transgenic TCR (DN1) in human CD1-transgenic mice offered a practical model to study CD1b-mediated mycolic acid–specific T-cell responses ^60^. In these mice, DN1 T cells rapidly localized to the lungs and adjacent mediastinal lymph nodes following aerosol challenge with *Mycobacterium tuberculosis* and provided detectable, albeit modest, protection from infection ^60^.

Because CD1b proteins are abundantly expressed on foam cells in human atherosclerotic plaques ^61^, Wang and colleagues asked whether cells expressing HJ1 might influence atherosclerosis in a model of accelerated vascular disease in mice lacking apolipoprotein E (apoE) ^62^. Unexpectedly, apoE knockout mice expressing the human CD1b transgene and the HJ1 TCR spontaneously developed a severe psoriaform dermatitis ^62^. The authors traced the disease to ectopic phospholipid deposition in skin, occurring as a result of hyperlipidemia. Intradermal HJ1-autoreactive T cells secreted IL-17 and IL-22, driving recruitment of neutrophils to lesional skin ^62^. The extent to which these events mimic the pathophysiology of human psoriasis is unknown. However, human psoriatic lesions overexpress CD1b as compared with normal skin, and there is evidence for altered lipids in psoriatic skin ^62, 63^.

## *In vivo* studies of CD1a in skin disease

**Figure 2.**
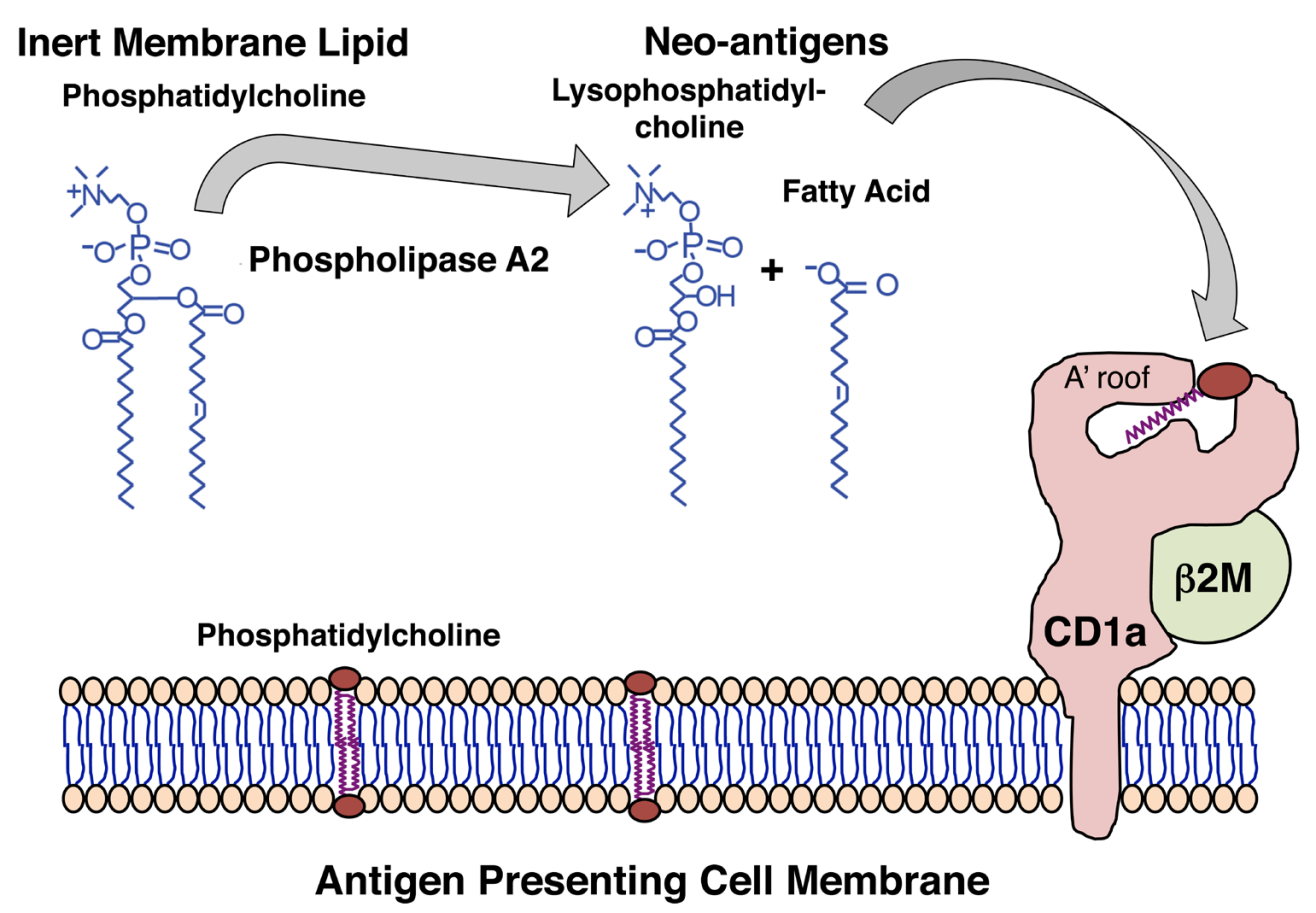
Model for generation of lipid neo-antigens from intact membrane lipids. The figure shows this through the action of phospholipases present in house dust mites ^66^ and bee and wasp venoms ^65, 67^ and viruses ^71^. Whereas cleavage of phosphatidylcholine is shown here, experimental evidence suggests that a similar mechanism could apply to other membrane phospholipids, including phosphatidylglycerol, phosphatidic acid, phosphatidylserine, and phosphatidylinositol. Newly generated lipid neo-antigens generated from self cellular lipids by phospholipases are thought to be loaded on CD1a through an unknown mechanism, where they can be presented to CD1a-reactive T cells.

Recent studies have suggested a role for human CD1a in psoriasis. In humans, enumeration of CD1a-autoreactive T cells *ex vivo* provided evidence for increased cell number in patients with psoriasis. This work ^64^, and related mechanistic studies ^65– 67^, revealed that CD1a-autoreactivity was associated with higher expression of lesional mast cell–derived phospholipase A _2_ (PLA _2_), which can convert non-antigenic phosphatidic acid–containing molecules into lysolipid neo-antigens ( Figure 2). Separately, a second-generation knockin mouse expressing the human CD1a transgene showed constitutive and cytokine-inducible CD1a expression at high levels ^68^. This mouse showed that CD1a expression markedly exacerbated skin inflammation in response to imiquimod-induced dermatitis, which mimics certain downstream events in psoriasis ^69^.

CD1a, which is constitutively expressed on epidermal Langerhans cells and is by far the most abundant CD1 antigen-presenting molecule in human skin, has recently been implicated in promoting other skin diseases. Whereas all humans show immune response to bee and wasp venoms, certain patients have severe local and systemic inflammation or anaphylaxis ^70^. In a recent study, Ogg and colleagues showed that bee and wasp venom–derived PLA _2_ released antigenic free fatty acids and lysophospholipids from non-antigenic phosphodiacylglycerol substrates ^65^. Interestingly, the PLA _2_-dependent activation of CD1a-reactive T cells requires both CD1a and a cellular membrane to provide lipid substrates. Hence, Langerhans cells may serve a dual role of antigen presentation by both providing CD1a and acting as a reservoir of membrane-tethered self-lipid antigen substrates. In this emerging model, wasp stings penetrate the skin to allow PLA enzymes to reach cellular phospholipid membranes and release self-lipids for catalysis to generate CD1a lipid neo-antigens ^65^.

Furthermore, both venom-allergic individuals and house dust mite–allergic individuals have a higher frequency of CD1a-reactive T cells that also release IFNγ, GM-CSF and IL-13 in response to PLA _2_^66, 67^. Desensitization immunotherapy for venom-allergic patients, consisting of serial subcutaneous injections of increasing doses of wasp venom over a course of 8 weeks, initially increases the frequency of IFNγ-expressing CD1a-reactive T cells in the first 3 to 5 weeks, which decreases by the end of treatment ^67^. Filaggrin is a skin-barrier protein, which blocks PLA _2_, and is thought to limit release of CD1a-reactive lipid neo-antigens ^66^. Finally, transgenic expression of human CD1a in the skin of mice strongly increases immune response to the allergenic compound, found in poison ivy, known as urushiol ^69^.

Combined, these recent clinically oriented studies offer candidate interventions to abrogate CD1a-mediated skin lesions. Antibodies that block PLA _2_, lipid neo-antigens, or CD1a itself might limit pathogenic activation of T cells in lesional skin. Because lipid agonists and antagonists can be readily formulated in skin creams, the potential for topical treatment of skin diseases can now be investigated.

## Designing lipid-based immunological therapies

The non-polymorphic nature of CD1 genes creates a situation in which nearly all humans express CD1 proteins with the same sequence and structure ^72^. As contrasted with highly polymorphic MHC-encoded genes, CD1-reactive T cells are not restricted to the genetic background of the donor. Thus, lipid ligands of CD1 proteins are attractive candidates as “one antigen for everyone” approaches for altering immune responses in humans.

Figure 1. highlights how the three modes of lipid recognition by T cells point toward distinct strategies to synthesize antigenic lipids such as immunomodulatory drugs that selectively alter human T-cell responses to CD1. The head group recognition model supports development of agonists or superagonists that mimic natural antigens but are chemically modified to increase, decrease, or alter the response of T cells ^73, 74^. This approach has been exploited to produce α-galactosyl ceramides with altered immunological properties (reviewed in 75). Absence of interference supports the development of lipids with large head groups that might be broadly acting antagonists of CD1-autoreactive T cells ^34, 37^. The altered CD1 model calls for agonists that are small lipids lacking head groups that readily load onto CD1 ^40^.

Moving from theory to practice, several related approaches are testing the concept that mycobacterial lipids could alter systemic responses to tuberculosis disease. These studies are based on evidence that *M. tuberculosis* expresses many lipid antigens to which CD1-reactive T cells respond ^76, 77^ and that transgenic expression of human CD1b in mice provides some mycobacterial containment *in vivo* ^60^. As discussed above, guinea pigs express homologs for human CD1a, CD1b, and CD1c ^50^, so they can serve as models to define vaccine-induced responses. Proof-of-concept studies show that immunization with mycolic acid, diacylated sulfoglycolipids, or phosphatidylinositol dimannosides reduced lung inflammation and provided some reduction in bacterial burden compared with unvaccinated animals ^52, 53^. However, vaccination with lipids alone did not surpass protection induced by Bacille Calmette-Guérin, the current tuberculosis vaccine, suggesting that further development is needed. Although most studies of immune response to vaccination emphasize peptide antigens, whole cell vaccines, like Bacille Calmette-Guérin, also contain lipid antigens, raising the possibility that generating lipid-specific immune responses occur during current vaccination approaches in ways that are not currently appreciated or measured ^78^.

## Summary

The discovery of CD1 antigen presentation pathways has provided a broader perspective about the natural targets of T cell response, which normally recognize both peptide and lipid antigens. The number of known CD1-presented antigens continues to increase, and three distinct molecular models for their recognition are emerging on the basis of strong structural and functional data ( Figure 1). Therefore, the key to harnessing this new basic insight that lipids are antigens for T cells will likely lie in generating better models for measuring the CD1-mediated T-cell response *in vivo* in animals and *ex vivo* in humans to understand and interrupt their disease-specific functions.
